# Isolation of *Metarhizium* spp. from rhizosphere soils of wild plants reflects fungal diversity in soil but not plant specificity

**DOI:** 10.1080/21501203.2018.1524799

**Published:** 2018-09-25

**Authors:** Oumi Nishi, Hiroki Sato

**Affiliations:** a Research Fellowship for Young Scientists, Japan Society for the Promotion of Science, Tokyo, Japan; b Forest Entomology Division, Forestry and Forest Products Research Institute, Tsukuba city, Japan

**Keywords:** *Metarhizium*, entomopathogens, rhizosphere competency, host specificity, PCR-RFLP, Hypocreales

## Abstract

Understanding the abundance and diversity of fungal entomopathogens associated with plant species is critical for improving their field efficacy as microbial insecticides. *Metarhizium* is a cosmopolitan entomopathogenic fungus, with some species in this genus showing rhizosphere competencies. This study sought to determine the abundance and diversity of *Metarhizium* spp. in rhizosphere soils of wild plants in a field in Japan. *Metarhizium* spp. were detected in 76.2% of 151 rhizosphere soil samples collected from 41 plant species using a plating method. The mean density of *Metarhizium* spp. in rhizosphere soils was 1.2 × 10^4^ colony forming units/g soil [base 10 logarithm of the mean = 4.06 (S.D. = 0.88)]. There was no significant difference in the densities and detection rates between Asteraceae and Poaceae as well as among two aster and one grass species. The fungal isolates were identified as five species, of which *M. pingshaense* was the most frequently detected and abundant species. No significant specific associations were recognised between the isolated *Metarhizium* spp. and the examined aster and grass species. Our findings demonstrated the high occurrence and abundance of *M. pingshaense* in rhizosphere soils of wild plants at the sampling site irrespective of host plant taxa.

## Introduction

Mitosporic fungi in the order Hypocreales (Ascomycota) are traditionally known for their entomopathogenic characteristics. *Akanthomyces lecanii* (previously known as *Lecanicillium lecanii), Beauveria bassiana* and *Metarhizium anisopliae* complex are well-known entomopathogens widely used for biological pest control in agriculture and forestry (Vega et al. 2012; Kepler et al. 2017). Recently, however, several species of these entomopathogens were shown to play multiple roles in nature as rhizosphere associates, endophytes, and possibly even plant growth-promoting agents (reviewed by St. Leger 2008; Vega 2008; Jaber and Ownley 2018).

The ascomycete genus *Metarhizium* (Hypocreales: Clavicipitaceae) is largely composed of entomopathogenic species that mostly produce green conidia on the corpses of their arthropod hosts, earning it the moniker “green muscardine fungus” (e.g. Kepler et al. 2014). *Metarhizium* spp. are generally found as anamorphs on more than 200 host species belonging to 17 families of Insecta and Acari (Roberts and St. Leger 2004; Zimmermann 2007). *Metarhizium* spp. are among the most abundant fungi isolated from soils, with their titres reaching 10^6^ colony forming units (CFUs)/g soil in grasslands (Milner 1992; Lomer et al. 2001); they are also closely associated with the plant rhizosphere (Hu and St. Leger 2002; Wyrebeck et al. 2011), can promote plant growth (Liao et al. 2014) and become established as root endophytes (Sasan and Bidochka 2012).

The current taxonomy of *Metarhizium* is based on molecular phylogenetic analyses of its DNA sequences because this genus includes many morphologically indistinguishable species (i.e. cryptic species). Ecological or physiological differences among most of these cryptic species remain unknown except for habitat types and thermal growth preferences (e.g. Driver et al. 2000; Bidochka et al. 2001; Nishi et al. 2013, 2017). Regarding their ecological aspects as rhizospheric fungi, specific associations between *Metarhizium* spp. and wild and cultivated plants have been reported in field studies in Canada and USA (Fisher et al. 2011; Wyrebek et al. 2011), but not in a study in Denmark (Steinwender et al. 2015). Additionally, isolation of *Metarhizium* spp. endophytically colonizing in wild plant roots showed a predominance of one species (Behie et al. 2015).

The ecology of *Metarhizium* as rhizospheric fungi remains poorly understood, partly because they are rarely isolated from rhizosphere soils of wild plant populations. In particular, little is known of *Metarhizium* spp. functioning as rhizospheric fungi in regions of Asia. To this end, in this study, we investigated both the abundance and diversity of *Metarhizium* spp. associated with wild plant rhizospheres in a site in Japan.

## Materials and methods

### Collection of root samples

Root samples were collected from the field site of the Forestry and Forest Product Research Institute (Matsunosato1, Tsukuba, Japan; 140°07ʹ32.6″E–140°08ʹ03.1″E, 36°00ʹ19.1″N–36°00ʹ41.5″N) in May and June 2014 and again in May 2015, using an established sampling grid, covering an area of approximately 900 m east to west and 750 m north to south (Supplementary Figure 1). The grid comprised 924 sampling sites (28 rows × 33 columns) established at 27-m intervals, which were located using both satellite photography and a hand-held global positioning system device (e-trec HC, Garmin, Switzerland). In 2014, 151 rhizosphere soil samples of 41 plants species in 21 families were collected (Table 1). In 2015, 33 soil samples of 3 plant species – annual bluegrass [*Poa annua* (Poaceae)]; Philadelphia fleabane [*Erigeron philadelphicus* (Asteraceae)]; Oriental false hawksbeard [*Youngia japonica* (Asteraceae)] – were collected (Table 2). In each sampling year, no more than one plant sample was collected of each plant species from a single site in the grid.

**Table 1.** Number of root samples collected in 2014.

Plant family	Nos. of plant species	Nos. of compartments	Nos. of root samples	
			*Metarhizium* was detected	Total
Apiaceae	1	1	1	1
Asteraceae	9	25	33	48
Boraginaceae	1	1	1	1
Brassicaceae	1	1	1	1
Campanulaceae	1	5	3	5
Caryophyllaceae	2	8	6	8
Convolvulaceae	1	1	1	1
Cupressaceae	1	1	1	1
Fabaceae	4	7	6	10
Iridaceae	1	2	2	2
Moraceae	1	1	1	1
Oxalidaceae	1	5	3	5
Papaveraceae	1	5	5	5
Pinaceae	1	1	0	1
Poaceae	8	25	30	37
Polygonaceae	1	6	6	6
Ranunculaceae	1	1	1	1
Rosaceae	2	5	4	5
Rubiaceae	1	1	0	1
Saururaceae	1	7	6	7
Vitaceae	1	3	4	4
Total	41	37	115	151

**Table 2.** Number of root samples of the three representative plant species.

Plant species (families)	Nos. of root samples	
	*Metarhizium* was detected	Total
Collection in 2014		
*Erigeron philadelphicus* (Asteraceae)	12	15
*Poa annua* subsp. *annua* (Poaceae)	15	15
*Youngia japonica* (Asteraceae)	10	16
Collection in 2015		
*Erigeron philadelphicus* (Asteraceae)	7	11
*Poa annua* subsp. *annua* (Poaceae)	9	15
*Youngia japonica* (Asteraceae)	4	7

Each plant sample was carefully uprooted after digging up the soil around its roots to a depth of approximately 10 cm. Soil weakly adhered to the roots was gently shaken off, and the root portion below 10 cm from the ground surface was cut off. All the root samples were individually contained in a clean plastic bag and kept in a cooler box before being preserved in a refrigerated chamber at 4°C. The root samples were processed for the isolation of *Metarhizium* spp. within 2 days of sampling.

### Isolation of *Metarhizium* spp. from root and rhizosphere soils

Each root sample was dissected to the length of approximately 1 cm and transferred into a sterile 50-mL centrifuge tube. More than 2 volumes of an aqueous solution of 0.05% Tween 80 were added to 1 volume of diced root samples in the centrifuge tube and mixed vigorously for 1 min on a vortex mixer. After removing residual root pieces from the suspension, a series of 3-fold serial dilutions ranging from 1/3 to 1/81 were made for the suspension, with 100 µl of each dilution spread onto a semi-selective agar plate (6% oatmeal flour, 1.25% agar, 0.1% cycloheximide, 0.03% chloramphenicol, Nishi et al. 2017). The plates were incubated at 25°C for at least 14 days. CFUs of visible *Metarhizium* spp. were counted and representatives of visibly different colonies in each soil sample were transferred to potato dextrose agar (PDA; 2.1% dextrose, 1.4% agar, 0.4% potato extract) plates.

### Quantification of densities of *Metarhizium* spp. in rhizosphere soil

To quantify the respective densities of *Metarhizium* spp. in rhizosphere soils (expressed as CFUs/g soil), the volumes and dry weight of each suspension were determined. Volume was measured using a measuring cylinder, and dry weight of soil was determined after the suspension was poured onto a tin tray and oven-dried at 180°C for 3 h. The mean and median of the dried weight of the 151 rhizosphere soil suspensions were 0.0063 and 0.0051 g/mL (S.D. = 0.0046), respectively.

A detection limit for the CFUs/g dried soil for each soil sample was designated as the values of CFUs/g soil when 1 CFU was detected on selective agar plates on which the most dense soil suspension of the serial dilutions was spread plated. The mean, median and standard deviation of density were obtained using the Kaplan–Meier estimates or from regression order statistics in the R package NADA, following Helsel (2011). Any zero values for density – when CFUs of *Metarhizium* were not detected – were treated as censored data with their detection limits as censored values.

Comparisons between two representative plant families, Asteraceae and Poaceae, were conducted with the samples collected in 2014. Comparisons among the three representative plant species (*P. annua, E. philadelphicus* and *Y. japonica*) were conducted with samples collected in 2014 and 2015. These particular families and species were used because they were widely distributed at the sampling site and occurred in relatively large numbers in our samples. For the 151 samples of 2014, the total densities of *Metarhizium* spp. were determined even when more than one *Metarhizium* sp. co-occurred with a single plant sample. For the 33 samples of 2015, the respective *Metarhizium* spp. densities were determined separately: all colonies of *Metarhizium* spp. observed on selective agar medium were transferred to PDA plates and grouped by their morphological characteristics of colonies, followed by species identification of each morphological type.

### Species identification of root isolates

Root isolates were first grouped according to their morphological characteristics and PCR-RFLP of the intergenic spacer region of rDNA (IGS) (Nishi et al. 2017). Species-level identifications of the representative isolates of these groups were conducted using a phylogenetic analysis of DNA sequences, in which the 5′-partial sequences of the translation elongation factor gene (5TEF) were analysed, as recommended by Bischoff et al. (2009). The species of the isolates other than the representative isolates were identified by the correspondence of species with either the morphological types or the PCR-RFLP genotypes.

Two distinct morphological groups recognised in the root isolates appeared to be *M. pemphigi* and *M. lepidiotae*, as confirmed by comparisons with Japanese *Metarhizium* isolates already identified by DNA sequences of 5TEF by Nishi et al. (2011). Three isolates per group were selected as representatives for the phylogenetic species identification. The other isolates were grouped by the PCR-RFLP of the IGS.

Crude DNA samples were prepared as follows: mycelia from a pure culture on an agar plate were picked up with a sterile micropipette tip and suspended in a 50-µl Tris-EDTA buffer containing RNase A [10 µmol/l pH 8.0 Tris-HCl, 1 µmol/l pH 8.0 EDTA, 0.01% RNase A (w/v)]. The suspensions were frozen at least once for the elution of DNA from the mycelia before being used for PCR. The crude DNA solutions were stored at −20°C.

PCRs of the IGS were performed in 10-µl reaction volumes, containing 0.1–10% (v/v) crude DNA solution, 1× reaction buffer for KOD FX Neo (TOYOBO, Japan), 0.5 µmol/l of each primer (Forward IGS28S4: CCTTGTTGTTACGATCTGCTGAGGG, Reverse IGS18S4: TAATGAGCCATTCGCAGTTTCGCTG, Pantou et al. 2003) and 0.1–0.2 U KOD FX Neo (TOYOBO). Amplification conditions were: 2 min at 94°C, followed by 40 amplification cycles for 10 s at 98°C, 30 s at 63°C and 2 min 30 s at 68°C, and final extension for 7 min at 68°C. PCR products of rDNA IGS were digested with *Hae*III (NEB, Japan). The restriction enzyme digestion was performed in volumes of 20 µl comprising 9 µl of PCR product, 1×restriction enzyme buffer, 1–3 units of the restriction enzyme and sterile distilled water. Reaction mixtures were placed in an incubator at 37°C for 12–16 h. Electrophoresis of 6–10 µl of each of the digested samples was performed on 2% Agarose 21 (Nippon Gene, Japan) using a 50-bp marker at 100 V for 30–60 min in 1×Tris-borate EDTA buffer. DNA fragments in the gel were visualised under ultraviolet (UV) light after gel staining with ethidium bromide (Supplementary Figure 2).

Species identification through a phylogenetic analysis of the DNA sequence of 5TEF was carried out as described by Nishi et al. (2017). *M. novazealandicum* ARSEF 3056 was used as the outgroup. The accession numbers of all DNA sequences are provided in Supplementary Table 1.

### Statistical analysis

Densities of propagules of *Metarhizium* spp. in rhizosphere soil (CFUs/g dried soil) were compared by the generalised Wilcoxon test, with *p*-values adjustment for multiple comparisons by the Bonferroni’s method. Detection rates of *Metarhizium* spp. in rhizosphere soil were compared with Fisher’s exact test, with *p*-values adjustment for multiple comparisons by the Benjamini and Hochberg’s method.

## Results

### Analysis of *Metarhizium* isolated from rhizosphere

*Metarhizium* spp. were detected in 76.2% (115/151) of the rhizosphere soil samples using the plating method. The estimated mean of *Metarhizium* spp. densities in the rhizosphere soils was 1.2 × 10^4^ CFUs/g dried soil [log-transformed value of the mean, 4.06 (S.D. = 0.88)]. The modal interval (10-fold interval) of this density was 1.0 × 10^4^ to 1.0 × 10^5^ CFUs/g dried soil. The top five densities were recorded from different plant species: common vetch [*Vicia sativa* subsp. *nigra* (Fabaceae)], 4.2 × 10^6^; annual bluegrass [*Poa annua* (Poaceae)], 9.0 × 10^5^; Japanese nipplewort [*Lapsana humilis* (Asteraceae)], 7.3 × 10^5^; sorrel [*Rumex acetosa* (Polygonaceae)], 6.5 × 10^5^ and oriental false hawksbeard [*Youngia japonica* (Asteraceae)], 6.5 × 10^4^ CFUs/g dried soil.

**Figure 1.**
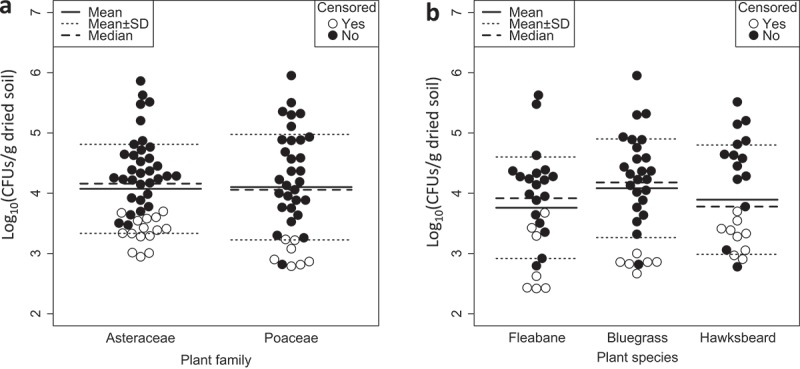
Density of *Metarhizium* spp. in rhizosphere soils of wild plants (log_10_ CFUs/g dried soil). (a). Comparison between two plant families (Asteraceae, *n* = 48; Poaceae, *n* = 37). (b). Comparisons among three plant species (Fleabane: *Erigeron philadelphicus* [Asteraceae], *n* = 26; Bluegrass: *Poa annua* subsp. *annua* [Poaceae] *n* = 30; Hawksbeard: *Youngia japonica* [Asteraceae], *n* = 23). Closed circles indicate the densities of *Metarhizium* spp. calculated from the CFUs counts actually observed on selective agar plates. Open circles indicate the detection limits of CFUs for non-detected samples (zero values were treated as censored data that are censored at their detection limits). Means, medians and standard deviations were estimated by ROS estimation in which zero values were censored at their detection limits. The densities were not significantly different among the plant families and species (*p* > 0.05; generalised Wilcoxon test).

The densities (log-transformed CFU/g dried soil) were not significantly different among the plant families and species (family, X^2^ = 0.3, d.f. = 1, *p* = 0.58; species, X^2^ = 1.5, d.f. = 2, *p* = 0.468, generalised Wilcoxon test, Figure 1(a,b)). Detection rates of the *Metarhizium* spp. were also not significantly different among the plant families and species (family, *p* = 0.22; species, *p* = 0.32, Fisher’s exact test; Table 3).

**Table 3.** Detection rates of *Metarhizium* spp. in rhizosphere soils of three species of wild plants.

	Detection rate (%)^a^				
Plant species	*M. lepidiotae*	*M. pemphigi*	*M. pingshaense*	*M. robertsii*	Total
*Erigeron philadelphicus* (*n* = 26)	15.4	3.8	61.5	15.4	73.1
*Poa annua* (*n* = 30)	20.0	0.0	63.3	33.3	80.0
*Youngia japonica* (*n* = 23)	8.7	4.3	47.8	30.4	60.9
*p*-value ^b^	0.54	0.52	0.51	0.16	0.32

^a^*M. guizhoense* was not isolated from the three plant species.

^b^ Fisher’s exact test

### Species identification of *Metarhizium* isolates from the rhizosphere

**Figure 2.**
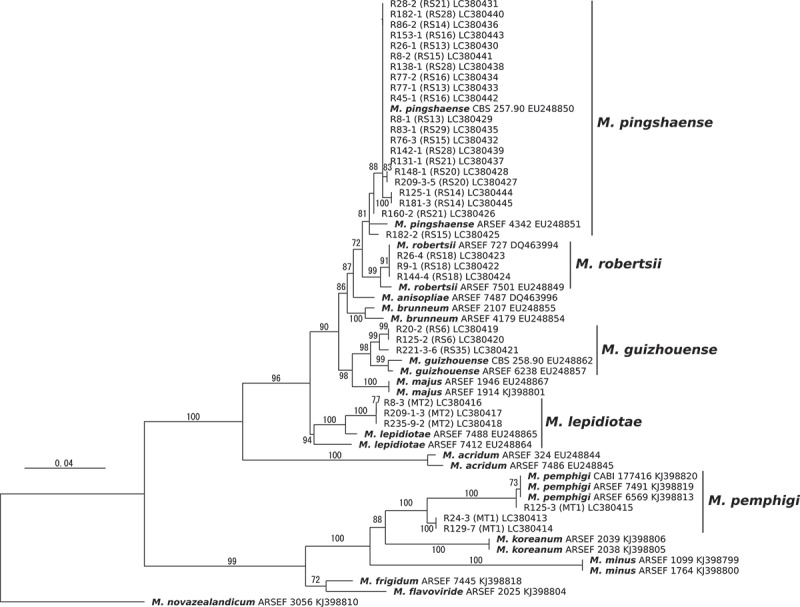
A maximum likelihood phylogeny inferred from the analysis of the 5′-partial sequences of the translation elongation factor gene of *Metarhizium* spp. isolated from rhizosphere soil. The support values obtained from 1,000 bootstrap replicates are presented above or below branches. The branch labels indicate isolate names, followed by the PCR-RFLP genotypes (RS) or the morphological types (MT1 or MT2) determined for the isolates in this study and genbank accession Nos. The branch labels with species names (bold font) were reference sequences for the species identification. *M. novazealandicum* ARSEF 3056 is an outgroup taxon.

Two distinct morphological groups were recognised in the isolates on the basis of morphological characteristics of colonies on PDA plates. Isolates of the morphological type (MT) 1 formed blight green conidia while those of MT 2 produced a fine mass of black and shiny conidia on a mycelial mat (Supplementary Figure 3). Three isolates each from MT1 and MT2 were, respectively, identified as *M. pemphigi* and *M. lepidiotae* in the phylogenetic analysis (Figure 2). According to this correspondence, 15 and 31 isolates were identified as *M. pemphigi* and *M. lepidiotae*, respectively.

Grouping by PCR-RFLP was performed for the other isolates. The size of PCR amplicons of the rDNA IGS region was approximately 2500–2800 bp. The RFLP obtained by digestion with HaeIII were grouped into 11 unique banding patterns (i.e. RS6, 13, 14, 15, 16, 18, 20, 21, 28, 29 and 35) (Supplementary Figure 2). Species identification by the DNA sequence of 5TEF was conducted for all isolates or 3 representatives from each RFLP group.

The phylogenetic analysis indicated that 3 isolates belonging to 2 genotypes (RS6 and 35); 21 isolates belonging to 8 genotypes (RS13, 14, 15, 16, 20, 21, 28 and 29) and 3 isolates belonging to RS18 were identified as *M. guizhouense, M. pingshaense*, and *M. robertsii*, respectively (Figure 2). Based on this correspondence between species and the PCR-RFLP genotype, a total of 3, 184 and 55 isolates were, respectively, identified as *M. guizhouense, M. pingshaense* and *M. robertsii*. The number of isolates identified as the 5 *Metarhizium* spp. was summarised in Supplementary Table 2.

### Comparing the detection rates among five *Metarhizium* spp

One and more than one species were detected in 49.0% (74/151) and 27.2% (41/151) of the total soil samples, respectively (Table 4). At most, three different species were detected from a single soil sample. *M. pingshaense* was included in 8 out of the 9 combinations of the co-occurrence. To detect a possible competitive interaction among the three major *Metarhizium* spp. (*M. lepidiotae, M. pingshaense* and *M. robertsii*), the independence among the detection frequencies of the three species was tested (the contingency tables for the analyses are presented as Supplementary Table 3, 4, 5). The results indicated negligible competitive effects among the three species (Fisher’s exact test, *M. lepidiotae*–*M. pingshaense, p* = 0.82; *M. lepidiotae*–*M. robertsii, p* = 1.00; *M. pingshaense*–*M. robertsii, p* = 0.55).

**Table 4.** Frequencies of the occurrence of a single or more than one *Metarhizium* spp. in a rhizosphere soil sample.

Detected species	*M. pingshaense*	*M. robertsii*	*M. lepidiotae*	*M. pemphigi*	*M. guizhouense*	Number of soil samples
One species	◯	–	–	–	–	55
	–	◯	–	–	–	8
	–	–	◯	–	–	7
	–	–	–	◯	–	4
Two species	◯	◯	–	–	–	17
	◯	–	◯	–	–	11
	◯	–	–	◯	–	2
	◯	–	–	–	◯	1
	–	◯	◯	–	–	3
Three species	◯	◯	◯	–	–	2
	◯	◯	–	◯	–	3
	◯	–	◯	◯	–	1
	◯	–	–	◯	◯	1
Not detected	–	–	–	–	–	36
Total						151

**Figure 3.**
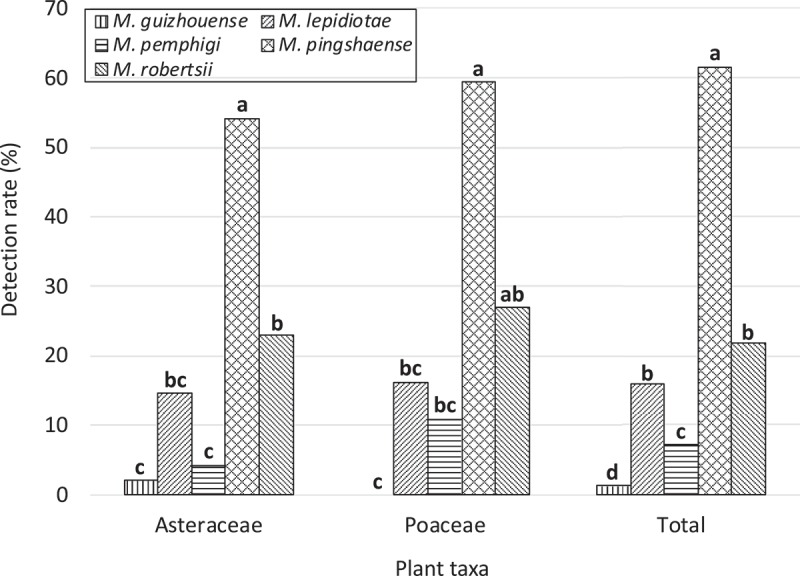
Detection rate of five *Metarhizium* spp. in rhizosphere soils of wild flowers (*n* = 151). Bars with the same letters are not significantly different (*p *> 0.01; multiple comparisons by Fisher’s exact test with Benjamini and Hochberg-adjusted *p* values).

Detection rates for the five *Metarhizium* spp. differed significantly for the two representative plant families as well as for the total sample (Asteraceae, *p* = 6.4 × 10^−11^; Poeaceae, *p* = 3.1 × 10^−9^; Total, *p* = 2.1 × 10^−16^, Fisher’s exact test; Figure 3). *M. pingshaense* was the most frequently detected species for Asteraceae and the total sample (*p* < 0.05, Fisher’s exact test with Benjamini and Hochberg-adjusted *p* values). Detection rates between the two families or among the three species were not significantly different for each of the five *Metarhizium* spp. (all *p* values > 0.05, Fisher’s exact test, Table 3).

### Comparing the density of three Metarhizium spp

Densities of each *Metarhizium* spp. were determined for the 33 rhizosphere soil samples from three plant species (bluegrass, fleabane and hawksbeard). From these, *Metarhizium* spp. were detected in 20 samples. The isolates were identified as *M. lepidiotae, M. pemphigi, M. pingshaense* and *M. robertsii*, which were detected from 6, 1, 18, and 8 root samples, respectively. *M. pemphigi, M. pingshaense* and *M. robertsii* were the most abundant species in 1, 15 and 4 samples, respectively. *M. pemphigi* was removed from the following comparisons because of its very low frequency.

In the analysis of individual three plant species, densities of *Metarhizium* detected from them were not significantly different (*M. lepidiotae, p* = 0.68, *M. pingshaense, p* = 0.45, *M. robertsii, p* = 0.18, generalised Wilcoxon test). Nonetheless, significant difference was recognised among the densities of three *Metarhizium* spp. in bluegrass and fleabane rhizosphere (bluegrass, *p* = 0.012; fleabane, *p* = 0.0067; hawksbeard, *p* = 0.7, generalised Wilcoxon test), but only so between *M. lepidiotae* and *M. pingshaense* for both plant species (bluegrass, *p* = 0.039; fleabane, *p* = 0.018; others: *p* > 0.05, generalised Wilcoxon test with Bonferroni-adjusted *p* values).

**Figure 4.**
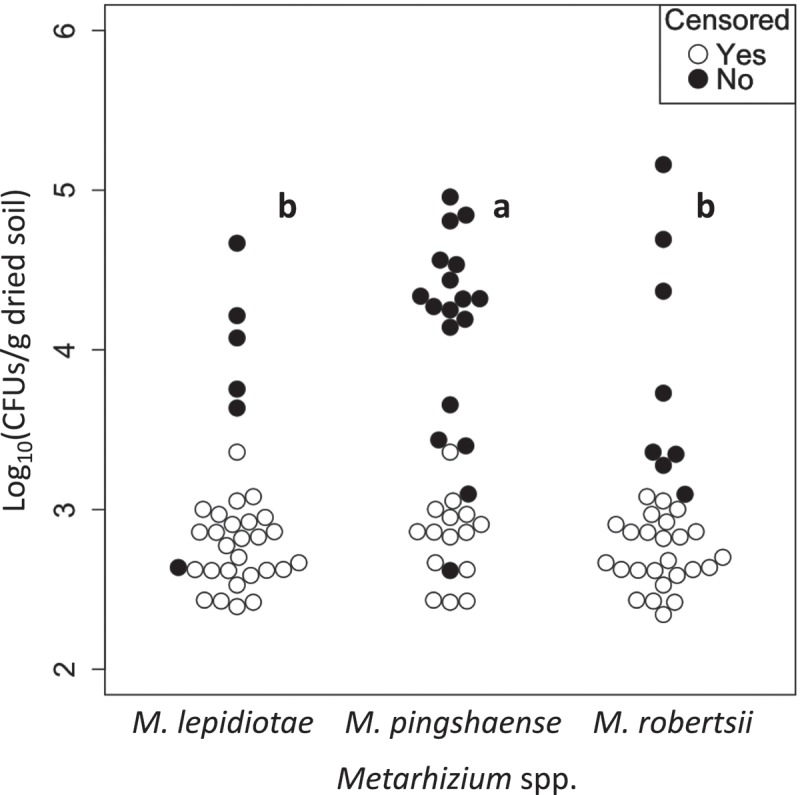
Comparisons of densities among the three *Metarhizium* spp. in rhizosphere soils of wild flowers (*n* = 33). Closed circles indicate the densities of *Metarhizium* spp. calculated from the number of CFUs observed on selective agar plates. Open circles indicate the detection limits of CFUs for non-detected samples (zero values were treated as censored data that are censored at their detection limits). Plot clusters with the same letters are not significantly different (*p *> 0.01; generalised Wilcoxon test with Bonferroni-adjusted *p* values).

In the analysis pooling all 33 root samples (Figure 4), densities of the three *Metarhizium* spp. significantly differed (*p* = 1.5 × 10^−4^, generalised Wilcoxon test) between *M. lepidiotae* and *M. pingshaense* and between *M. pingshaense* and *M. robertsii* (*M. lepidiotae*–*M. pingshaense, p* = 7.2 × 10^−4^; *M. pingshaense*–*M. robertsii, p* = 0.012, generalised Wilcoxon test with Bonferroni-adjusted *p* values).

## Discussion

*Metarhizium* spp. are ubiquitous in soil microbial communities, attaining levels that average 1.0 × 10^3^ CFUs/g soil, with a maximum of 1.0 × 10^6^ CFUs/g soil, without a special reference to the rhizosphere (Milner 1992; Lomer et al. 2001; Scheepmaker and Butt 2010). *Metarhizium* spp. were also found to maintain greater abundance levels in the inner than outer rhizosphere soil in a field of cabbages (Hu and St Leger 2002). Although close associations of three *Metarhizium* spp. to wild plants rhizospheres were reported in field studies in eastern Canada (Wyrebek et al. 2011; Behie et al. 2015), surprisingly little has been reported on *Metarhizium* abundance levels in the rhizospheres of wild plant species. In this study, we obtained a mean and maximum density of *Metarhizium* in the rhizospheres of wild plants at our sampling site of 1.2 × 10^4^ and 4.2 × 10^6^ CFUs/g dried soil, respectively. These values appear higher than the *Metarhizium* densities in soils without special reference to rhizosphere reported in previous studies. This result also suggests that root herbivores in this studied field site are generally exposed to *Metarhizium* spp., and they may be infected by the root-associated *Metarhizium* according to Kyeser et al. (2014). At this density, *Metarhizium* spp. could reduce populations of some root herbivore insects, given the dose of *Metarhizium* spp. required to kill white grubs that damage sugar cane roots; the LC_50_ value of *M. anisopliae sensu lato* F-1045 tested against a cane grub species was 8.7 × 10^4^ conidia/g peat, while a dose of 1 × 10^6^ conidia/g peat killed 96% and 85% of two sugar cane grub species (Milner et al. 2010).

Specific rhizosphere associations reportedly occur in some ectomycorrhizal fungi and woody plants (Bruns et al. 2002; Sato 2016). Arbuscular mycorrhizal fungi can also show ecological affinities to herbaceous plant species, including plants of Poaceae and Asteraceae, although this fungal group is largely not host-specific (Klironomos 2000; Santos-Gonzalez et al. 2006; Torrecillas et al. 2012). *Metarhizium* spp. have been reported to show specific associations with plant rhizospheres in two field studies in Canada and USA (Fisher et al. 2011; Wyrebek et al. 2011). However, the results of this study did not find support for a specific association between *Metarhizium* spp. and plant species of Poaceae and Asteraceae, which was similar to the results observed for *Metarhizium* spp. in crop rhizosphere soils in an agricultural field in Denmark (Steinwender et al. 2015). These inconsistent results may be due to the differences in the composition of *Metarhizium* spp. distributed among these sampling sites and the particular plant species used in the focussed comparisons. For example, while *M. brunneum* showed specific associations in the two North American studies (Canada and USA), this species was not isolated from our sampling site. Moreover, *Metarhizium* species from different populations may associate differently with plant species; for example, an *M. brunneum* population in Canada was associated with the rhizosphere of woody plants (shrubs and trees), whereas its USA counterpart was associated with strawberries and blueberries rather than Christmas trees.

Furthermore, environmental factors can influence the spatial distributions of species. Thus various factors other than plant species should be considered when assessing the specificity or preference of *Metarhizium* spp. against plant species. Wyrebek et al. (2011) have suggested that *Metarhizium* spp. in a field in Ontario, Canada, were associated with plant groups such that *M. robertsii* and *M. brunneum* were associated with the rhizospheres of grasses and woody plants (trees and shrubs), respectively. These associations may reflect differences in a thermal growth preferences and a resilience to UV radiation rather than adaptation to plant species per se because *M. robertsii* isolated from soil in that area did prefer a higher temperature and had higher resilience to UV radiation compared with *M. brunneum* (Bidochka et al. 2001; Bischoff et al. 2009) as well as because woody plant rhizosphere soils likely have lower temperatures and receive weaker UV radiation than do grass rhizospheres due to pronounced differences in direct solar irradiance. To support their view, the authors provided data showing that *M. robertsii* conidia germinated significantly better in switchgrass root exudate than *M. brunneum* conidia, but germination rates in woody plant root exudates were not shown. In work by Fisher et al. (2011), locations rather than plant species may have determined the detection rate of *Metarhizium* spp. They demonstrated that the *Metarhizium* species composition from Christmas tree rhizospheres at their sampling site markedly differed from the species composition of three other plant species; however, their Christmas tree sampling sites were restricted to a smaller area than used for the other plant species. Although little experimental evidence was found for specific associations of *Metarhizium* spp. with plant species, Liao et al. (2014) have demonstrated that an *M. brunneum* strain colonised corn roots just as good as a *M. robertsii* strain over a 3-month experimental period. They suggested that the natural distributions of these fungi do not necessarily predict their persistence in rhizosphere soil after artificial introductions, at least in the short term. Thus, for further inquiry into the specificity or preference of *Metarhizium* spp. for plant species, it seems necessary to conduct multivariate analyses including key environmental factors of rhizosphere soils, similar to analyses in studies by Rath et al. (1992) and Quesada-Moraga et al. (2007), coupled with inoculation assays over long monitoring periods.

Our field data demonstrated that *M. pingshaense* was the dominant species in the rhizospheres among the five *Metarhizium* spp. (Figures 3, 4). Similarly, previous studies have reported *M. pingshaense* as the most frequently isolated species from non-rhizospheric soils and insects in Japan (Nishi et al. 2011; Nishi and Sato 2017). A plausible reason for such a high prevalence of *M. pingshaense* in rhizosphere soils may be its better ability to colonise roots. Nonetheless, a broad host range of *M. pingshaense* as an insect pathogen (e.g. Nishi and Sato 2017) may have also contributed to its high prevalence because this would provide greater opportunities to infect and produce conidia on insects inhabiting the rhizospheres, leading to the accumulation of *M. pingshaense* in both rhizospheric and non-rhizospheric soils. Our study also discovered that *M. pingshaense* isolated from the rhizospheres displayed larger variation in its IGS region compared with *M. robertsii* and *M. guizhouense*. Two IGS genotypes, RS28 and RS29, were not found in the 302 soil isolates of *Metarhizium* obtained from across Japan (Nishi et al. 2017) and were identified for the first time in the present study. Contradictory to our results, *M. robertsii* was the most prevalent and genetically diverse *Metarhizium* species in an agricultural field in the USA (Kepler et al. 2015). *M. robertsii* was also the predominant species among three *Metarhizium* spp. occurring as root endophytes of wild plants in Canada (Behie et al. 2015). Both *M. pingshaense* and *M. robertsii* are sister species and similar to each other in terms of pathogenicity (i.e. both have wide host range, e.g. Bischoff et al. 2009; Nishi and Sato 2017) and thermal growth characteristics (i.e. both are relatively adapted to higher temperature, e.g. Nishi et al. 2013). The fact that these two species did not significantly differ in their associations with plant species supports their ecological equivalence.

Co-occurrences of more than one species or genotypes of *Metarhizium* in a single root sample were frequently detected in this study (Table 4). The independence of the detection frequencies found for the three majority species (*M. lepidiotae, M. pingshaense* and *M. robertsii*) suggests a low competition among these species; however, Wyrebek et al. (2011) have suggested that competition could occur between two *Metarhizium* spp. in root colonisation. Our results suggest that the three *Metarhizium* spp. can persist in the same rhizosphere environment when they are co-introduced to soils. Occupying different spatial niches at microhabitat scale in a root system may enable the coexistence. Little is known regarding the spatial distributions of *Metarhizium* spp. in the rhizosphere except for *M. robertsii*: an isolate of *M. robertsii* most effectively colonised at a depth of 0–2 cm in cabbage roots and endophytically colonised the cortical cells of bean roots (Hu and St Leger 2002; Sasan and Bidochka 2012). In light of these features of *M. robertsii*, further investigations that detail the spatial distributions of *Metarhizium* species in the wild plant rhizospheres may be beneficial for understanding the mechanisms of their co-occurrence.

In conclusion, our findings in this study demonstrate the high occurrence and abundance of *M. pingshaense* in the rhizospheres of wild plants at the sampling site in Japan irrespective of plant taxa, which suggests *M. pingshaense* may perform better in terms of colonising the rhizospheres of various plant species. Field studies that directly consider various environmental factors and inoculation assays will help improve our understanding of the ecological features of *Metarhizium* spp. as rhizospheric fungi.
