# Supplementary material for: Isolation of Metarhizium spp. from rhizosphere soils of wild plants reflects fungal diversity in soil but not plant specificity
Source: Mycology. 2018 Sep 25;10(1):22–31. doi: 10.1080/21501203.2018.1524799 (PMC6394287; doi:10.1080/21501203.2018.1524799)
Supplement: Supplemental Material [file TMYC_A_1524799_SM7387.zip › Supplementary_Figures1-3.pdf]

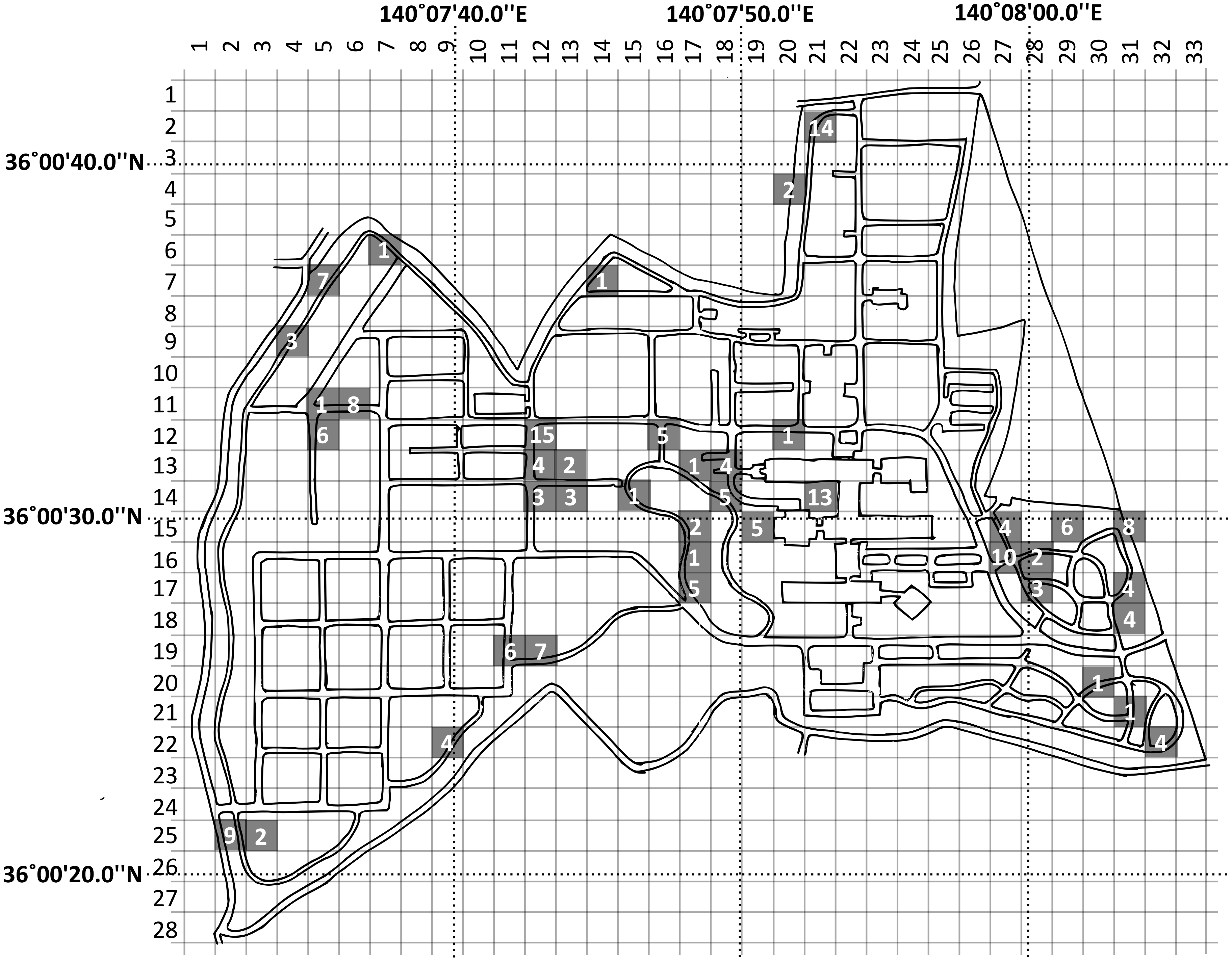

Supplementary Figure 1. Sampling area. Plant samples were collected from the compartments with grey shade. The numbers of plant samples collected in 2014 in each compartment are presented in the grey shade.

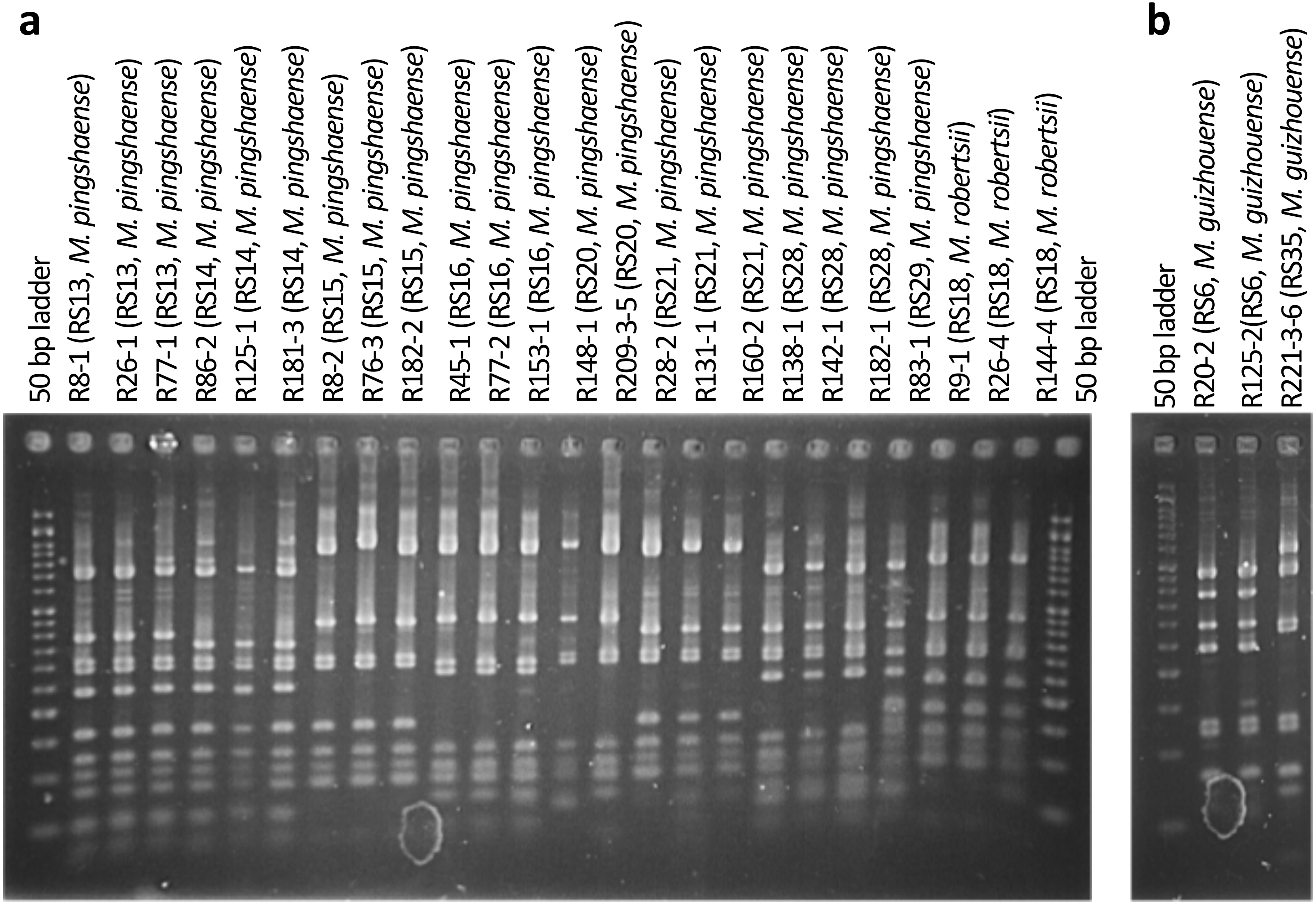

Supplimentary Figure 2. PCR-RFLP profiles of *Metarhizium* isolates from rhizosphere soil. PCR–RFLP profiles of *Metarhizium* spp. (amplicons of rDNA IGS digested with *Hae*III). a. Profiles of 24 isolates that are classified into 9 genotypes (RS13–16, 18, 21, 22, 28, 29). b. Profiles of 3 isolates that classified into 2 genotypes (RS6, 35). The size marker consists of 17 fragments between 50 and 1,200 bp (every 50 bp up to 500 bp, every 100 bp from 500 bp to 1,000 bp, 1,200 bp, and 1,500 bp). The characters after the isolate names indicate the genotypes of PCR–RFLP and species identified by the 5TEF sequences.

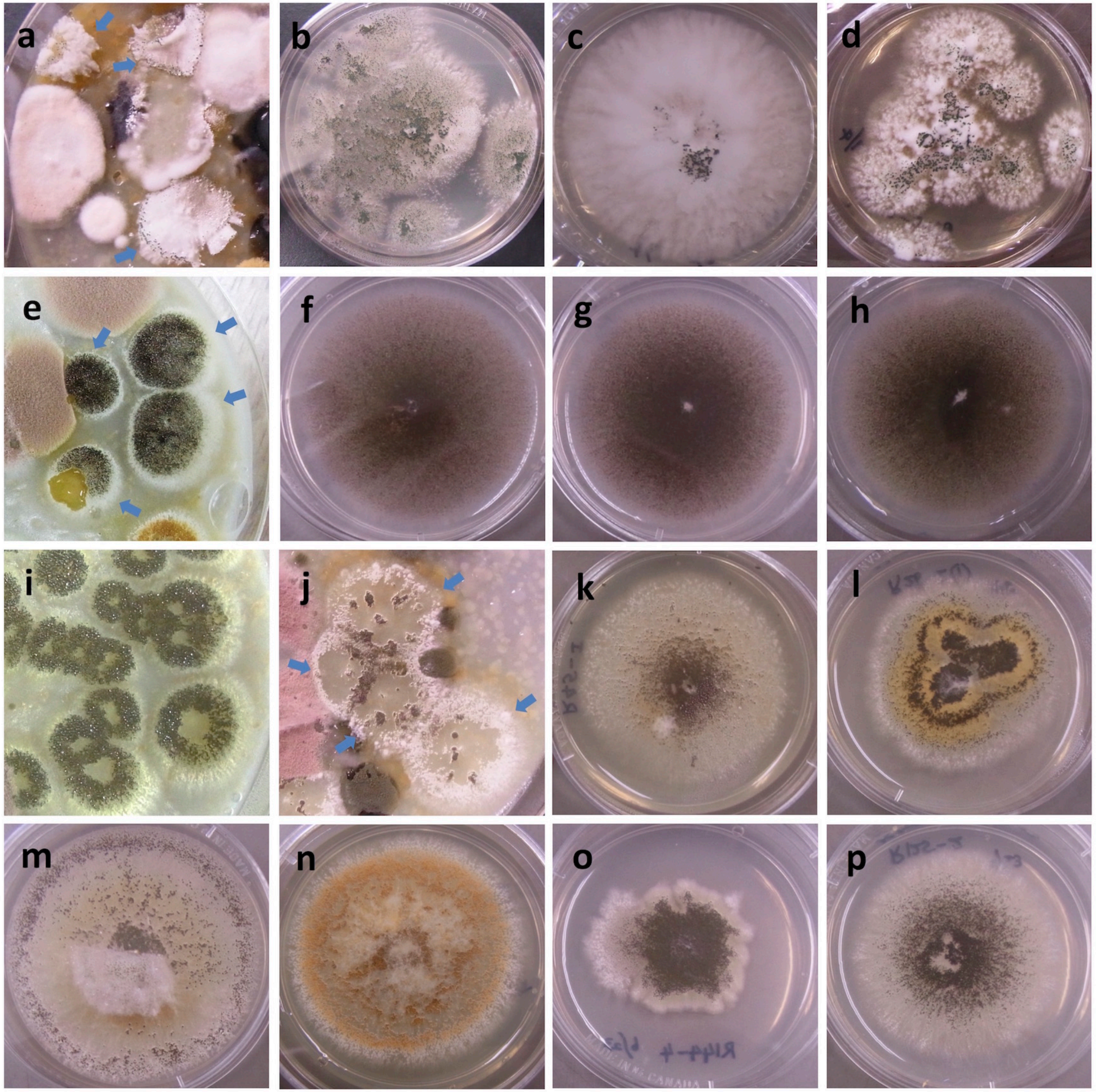

Supplementary Fig. 3 Colony morphology of *Metarhizium* spp. isolated from wild plant rhizosphere soils. **a**-**d**: morphological type (MT) 1; **e**-**h**: MT 2; **i**-**p**: the other MTs. **a**, **e**, **i**, **j**: colonies formed on the oatmeal-based semi selective agar medium (arrowed). **b**-**d**, **f**-**h**, **k**-**p**: colonies formed on the potato dextrose agar medium.
