# Supplementary material for: Isolation of Metarhizium spp. from rhizosphere soils of wild plants reflects fungal diversity in soil but not plant specificity
Source: Mycology. 2018 Sep 25;10(1):22–31. doi: 10.1080/21501203.2018.1524799 (PMC6394287; doi:10.1080/21501203.2018.1524799)
Supplement: Supplemental Material [file TMYC_A_1524799_SM7387.zip › Supplementary_Table_R1.docx]

Supplementary Table 1 *Metarhizium* isolates selected as representative of the respective PCR-RFLP genotypes or morphological types.

| RFLP genotypes (RS) or morphological types (MT) | Representative isolates | 5TEF  Accession No. | Species |
| --- | --- | --- | --- |
| RS6 | R20-2 | LC380419 | *M. guizhouense* |
|  | R125-2 | LC380420 | *M. guizhouense* |
| RS13 | R8-1 | LC380429 | *M. pingshaense* |
|  | R26-1 | LC380430 | *M. pingshaense* |
|  | R77-1 | LC380433 | *M. pingshaense* |
| RS14 | R86-2 | LC380436 | *M. pingshaense* |
|  | R125-1 | LC380444 | *M. pingshaense* |
|  | R181-3 | LC380445 | *M. pingshaense* |
| RS15 | R8-2 | LC380441 | *M. pingshaense* |
|  | R76-3 | LC380432 | *M. pingshaense* |
|  | R182-2 | LC380425 | *M. pingshaense* |
| RS16 | R45-1 | LC380442 | *M. pingshaense* |
|  | R77-2 | LC380434 | *M. pingshaense* |
|  | R153-1 | LC380443 | *M. pingshaense* |
| RS18 | R9-1 | LC380422 | *M. robertsii* |
|  | R26-4 | LC380423 | *M. robertsii* |
|  | R144-4 | LC380424 | *M. robertsii* |
| RS20 | R148-1 | LC380428 | *M. pingshaense* |
|  | R209-3-5 | LC380427 | *M. pingshaense* |
| RS21 | R28-2 | LC380431 | *M. pingshaense* |
|  | R131-1 | LC380437 | *M. pingshaense* |
|  | R160-2 | LC380426 | *M. pingshaense* |
| RS28 | R138-1 | LC380438 | *M. pingshaense* |
|  | R142-1 | LC380439 | *M. pingshaense* |
|  | R182-1 | LC380440 | *M. pingshaense* |
| RS29 | R83-1 | LC380435 | *M. pingshaense* |
| RS35 | R221-3-6 | LC380421 | *M. guizhouense* |
| MT1 | R24-3 | LC380413 | *M. pemphigi* |
|  | R125-3 | LC380415 | *M. pemphigi* |
|  | R129-7 | LC380414 | *M. pemphigi* |
| MT2 | R8-3 | LC380416 | *M. lepidiotae* |
|  | R209-1-3 | LC380417 | *M. lepidiotae* |
|  | R235-9-2 | LC380418 | *M. lepidiotae* |

Supplementary Table 2. Number of isolates identified as the five *Metarhizium* spp.

| **Species** | **Number of isolates** | |
| --- | --- | --- |
|  | **Total** | **5TEF sequence was determined** |
| ***M. guizhouense*** | 3 | 3 |
| ***M. lepidioae*** | 31 | 3 |
| ***M. pemphigi*** | 15 | 3 |
| ***M. pingshaense*** | 184 | 21 |
| ***M. robertsii*** | 55 | 3 |
| **Total** | 288 | 33 |

Supplementary Table 3. The contingency table of the detection frequencies of *M. pingshaense* and *M. robertsii*. The numbers indicate the count of soil samples from which each species was detected or not detected.

|  | | *M. pingshaense* | |
| --- | --- | --- | --- |
|  |  | Detected | Not detected |
| *M. robertsii* | Detected | 22 | 11 |
|  | Not detected | 71 | 47 |

Supplementary Table 4. The contingency table of the detection frequencies of *M. lepidiotae* and *M. pingshaense*. The numbers indicate the count of soil samples from which each species was detected or not detected.

|  | | *M. pingshaense* | |
| --- | --- | --- | --- |
|  |  | Detected | Not detected |
| *M. lepidiotae* | Detected | 14 | 10 |
|  | Not detected | 79 | 48 |

Supplementary Table 5. The contingency table of the detection frequencies of *M. lepidiotae* and *M. robertsii*. The numbers indicate the count of soil samples from which each species was detected or not detected.

|  | | *M. robertsii* | |
| --- | --- | --- | --- |
|  |  | Detected | Not detected |
| *M. lepidiotae* | Detected | 5 | 19 |
|  | Not detected | 28 | 99 |
